# Rare case of congenital pterygium involving the right eye: clinical image

**DOI:** 10.11604/pamj.2026.53.14.50387

**Published:** 2026-01-13

**Authors:** Aarti Gopal Kute, Amol Deshpande

**Affiliations:** 1 Department of Rachana Sharir, Mahatma Gandhi Ayurved College Hospital and Research Centre, Datta Meghe Institute of Higher Education and Research (Deemed to be University), Salod (H), Wardha, Maharashtra, India

**Keywords:** Congenital pterygium, corneal involvement, ocular surface lesion

## Image in medicine

A 25-year-old male presented with a congenital pterygium involving the nasal aspect of the right eye. The lesion appears as a fibrovascular fold of conjunctival tissue extending from the nasal canthus onto the peripheral cornea. The surface is smooth and non-inflamed, with no evidence of active vascular congestion or scarring. The corneal involvement is limited, and the visual axis remains clear. Congenital pterygium is an exceptionally rare ocular anomaly, distinct from the more common acquired form typically associated with chronic ultraviolet exposure. The patient had no history of ocular trauma, irritation, surgery, or systemic abnormalities. Visual acuity was preserved, and the remainder of the ocular examination was unremarkable. Given the absence of symptoms and lack of progression, conservative management with periodic observation was recommended. This image highlights the characteristic appearance of a rare congenital pterygium.

**Figure 1:**
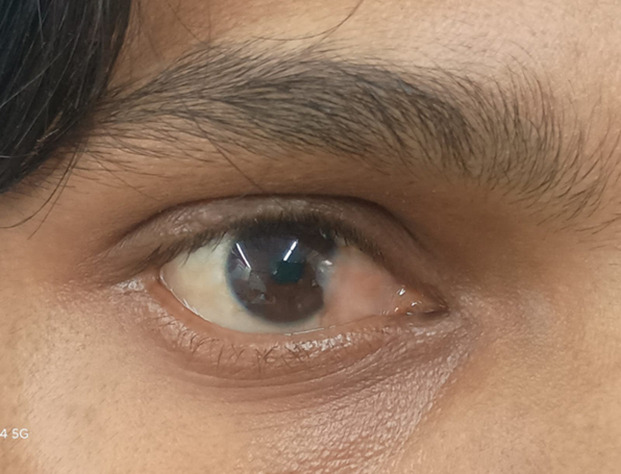
right eye showing a congenital fibrovascular pterygium extending from the nasal conjunctiva onto the central cornea with associated stromal opacity but preserved visual axis clarity

